# Supplementary figures and images for: Insecticidal activity of Ageratina adenophora (Asteraceae) extract against Limax maximus (Mollusca, Limacidae) at different developmental stages and its chemical constituent analysis
Source: PLoS One. 2024 Apr 16;19(4):e0298668. doi: 10.1371/journal.pone.0298668 (PMC11020717; doi:10.1371/journal.pone.0298668)

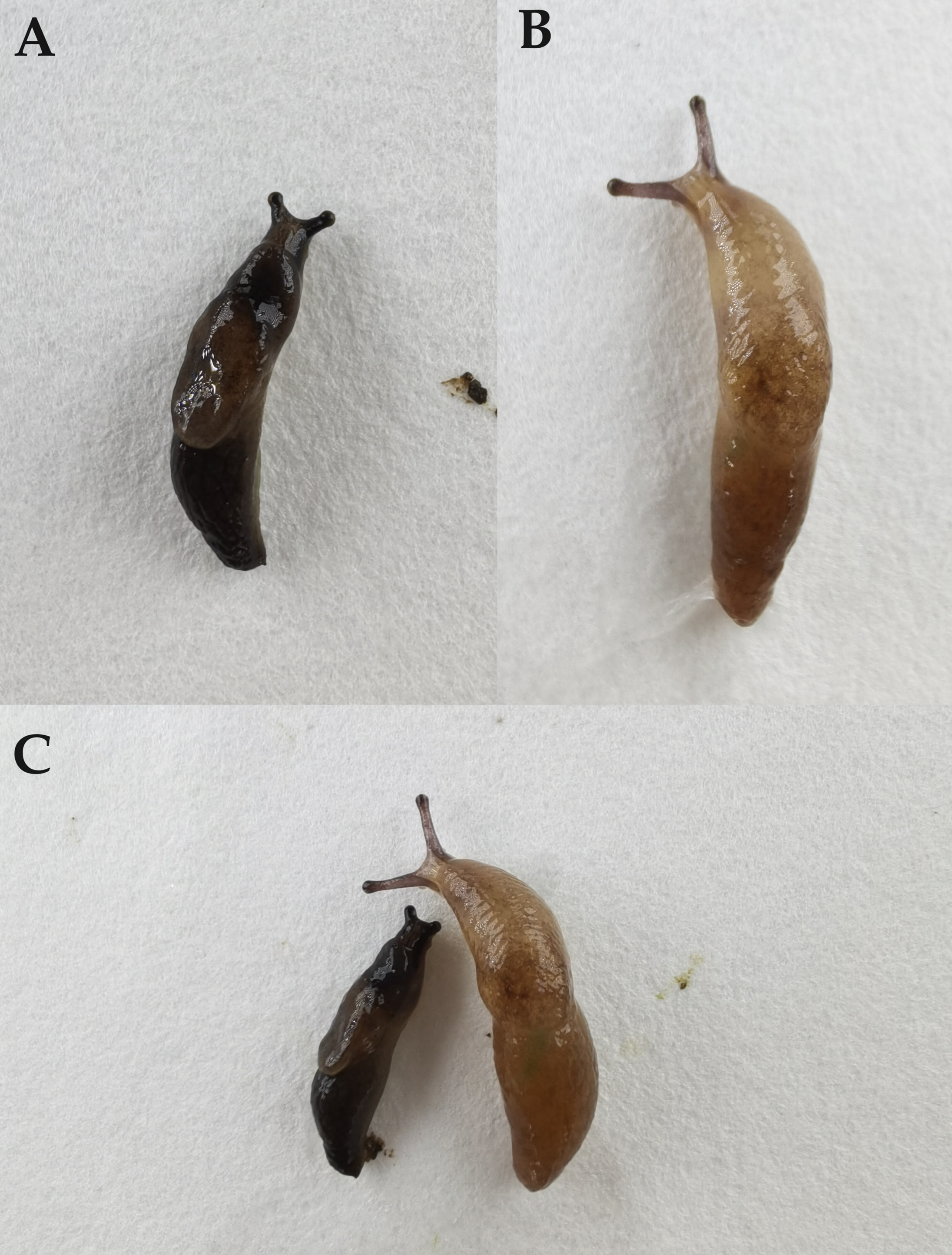

Supplement: S1 Fig — (A):Control; (B):Exposed; (C):Compare. (TIF) [file pone.0298668.s001.tif]
