# Supplementary material for: Insecticidal activity of Ageratina adenophora (Asteraceae) extract against Limax maximus (Mollusca, Limacidae) at different developmental stages and its chemical constituent analysis
Source: PLoS One. 2024 Apr 16;19(4):e0298668. doi: 10.1371/journal.pone.0298668 (PMC11020717; doi:10.1371/journal.pone.0298668)
Supplement: Attachment — Submitted filename: PONE-D-23-33974_reviewed.pdf [file pone.0298668.s003.pdf]

PLOS ONE

Insecticidal activity of Ageratina adenophora (Asteraceae) extract against Limax maximus (Mollusca, Limacidae) at different developmental stages and its chemical constituent analysis

--Manuscript Draft--

Manuscript Number:	PONE-D-23-33974
Article Type:	Research Article
Full Title:	Insecticidal activity of Ageratina adenophora (Asteraceae) extract against Limax maximus (Mollusca, Limacidae) at different developmental stages and its chemical constituent analysis
Short Title:	Insecticidal activity of Ageratina adenophora extract against Limax maximus at different developmental stages
Corresponding Author:	haojun li<br>Guizhou University College of Agriculture<br>guiyang, CHINA
Keywords:	Limax maximus, Ageratina adenophora, molluscide, agricultural pest control, plant extract
Abstract:	Limax maximus, or great gray slug, is a common pest in agriculture. The pest infests crops during their growth phase, creating holes in vegetable leaves, particularly in seedlings and tender leaves. A study was conducted to assess the insecticidal activity of Ageratina adenophora extract against these slugs. Factors such as fecundity, growth, hatching rate, offspring survival rate, protective enzyme activity, and detoxifying enzyme activity were examined in slugs exposed to the sublethal concentration (LC50) of the extract for two different durations (24 and 48 h). The phytochemical variability of the extracts was also studied. The LC50 value of the A. adenophora extract against L. maximus was found to be 35.9 mg/mL. This extract significantly reduced the hatching rate of eggs and the survival rate of offspring hatched from exposed eggs compared with the control. The lowest rates were observed in those exposed for 48 h. The survival, growth, protective enzyme activity, and detoxification activity of both newly hatched and 40-day-old slugs decreased. The A. adenophora extract contained tannins, flavonoids, and saponins, which may contribute to their biological effects. These results suggest that the extract could be used as an alternative treatment for slug extermination, effectively controlling this species.
Order of Authors:	haojun li

Powered by Editorial Manager® and ProduXion Manager® from Aries Systems Corporation

	runa zhao
	yingna pan
	hui tian
	wenlong chen
Additional Information:	
Question	Response
Financial Disclosure<br><br>Enter a financial disclosure statement that describes the sources of funding for the work included in this submission. Review the submission guidelines for detailed requirements. View published research articles from PLOS ONE for specific examples.	The author(s) received no specific funding for this work.

This statement is required for submission and will appear in the published article if the submission is accepted. Please make sure it is accurate.<br><br>Funded studies<br>Enter a statement with the following details:<br>• Initials of the authors who received each award<br>• Grant numbers awarded to each author<br>• The full name of each funder<br>• URL of each funder website<br>• Did the sponsors or funders play any role in the study design, data collection and analysis, decision to publish, or preparation of the manuscript?<br><br>Did you receive funding for this work?	

Competing Interests<br><br>Use the instructions below to enter a competing interest statement for this submission. On behalf of all authors, disclose any competing interests that could be perceived to bias this work—acknowledging all financial support and any other relevant financial or nonfinancial competing interests.<br><br>This statement is required for submission and will appear in the published article if the submission is accepted. Please make sure it is accurate and that any funding sources listed in your Funding Information later in the submission form are also declared in your Financial Disclosure statement.<br><br>View published research articles from PLOS ONE for specific examples.	The authors have declared that no competing interests exist.

NO authors have competing interests<br><br>Enter: The authors have declared that no competing interests exist.<br><br>Authors with competing interests<br><br>Enter competing interest details beginning with this statement:<br><br>I have read the journal's policy and the authors of this manuscript have the following competing interests: [insert competing interests here]<br><br>* typeset	

Powered by Editorial Manager® and ProduXion Manager® from Aries Systems Corporation

Ethics Statement<br><br>Enter an ethics statement for this submission. This statement is required if the study involved:<br><br>• Human participants<br>• Human specimens or tissue<br>• Vertebrate animals or cephalopods<br>• Vertebrate embryos or tissues<br>• Field research<br><br>Write "N/A" if the submission does not require an ethics statement.<br><br>General guidance is provided below. Consult the submission guidelines for detailed instructions. Make sure that all information entered here is included in the Methods section of the manuscript.	Provincial Key Laboratory for Agricultural Pest Management of Mountainous Region, Institute of Entomology, Guizhou University

Powered by Editorial Manager® and ProduXion Manager® from Aries Systems Corporation

Format for specific study types<br><br>Human Subject Research (involving human participants and/or tissue)<br>• Give the name of the institutional review board or ethics committee that approved the study<br>• Include the approval number and/or a statement indicating approval of this research<br>• Indicate the form of consent obtained (written/oral) or the reason that consent was not obtained (e.g. the data were analyzed anonymously)<br><br>Animal Research (involving vertebrate animals, embryos or tissues)<br>• Provide the name of the Institutional Animal Care and Use Committee (IACUC) or other relevant ethics board that reviewed the study protocol, and indicate whether they approved this research or granted a formal waiver of ethical approval<br>• Include an approval number if one was obtained<br>• If the study involved non-human primates, add additional details about animal welfare and steps taken to ameliorate suffering<br>• If anesthesia, euthanasia, or any kind of animal sacrifice is part of the study, include briefly which substances and/or methods were applied<br><br>Field Research<br><br>Include the following details if this study involves the collection of plant, animal, or other materials from a natural setting:<br>• Field permit number<br>• Name of the institution or relevant body that granted permission	
Data Availability<br><br>Authors are required to make all data underlying the findings described fully available, without restriction, and from the time of publication. PLOS allows rare exceptions to address legal and ethical concerns. See the PLOS Data Policy and FAQ for detailed information.	Yes - all data are fully available without restriction

A Data Availability Statement describing where the data can be found is required at submission. Your answers to this question constitute the Data Availability Statement and will be published in the article, if accepted.<br><br>Important: Stating ‘data available on request from the author’ is not sufficient. If your data are only available upon request, select ‘No’ for the first question and explain your exceptional situation in the text box.<br><br>Do the authors confirm that all data underlying the findings described in their manuscript are fully available without restriction?	

Describe where the data may be found in full sentences. If you are copying our sample text, replace any instances of XXX with the appropriate details.<br><br>• If the data are held or will be held in a public repository, include URLs, accession numbers or DOIs. If this information will only be available after acceptance, indicate this by ticking the box below. For example: All XXX files are available from the XXX database (accession number(s) XXX, XXX.).<br>• If the data are all contained within the manuscript and/or Supporting Information files, enter the following: All relevant data are within the manuscript and its Supporting Information files.<br>• If neither of these applies but you are able to provide details of access elsewhere, with or without limitations, please do so. For example:<br><br>Data cannot be shared publicly because of [XXX]. Data are available from the XXX Institutional Data Access / Ethics Committee (contact via XXX) for researchers who meet the criteria for access to confidential data.<br><br>The data underlying the results presented in the study are available from (include the name of the third party	All relevant data are within the manuscript and its Supporting Information files.

and contact information or URL).<br>• This text is appropriate if the data are owned by a third party and authors do not have permission to share the data.<br><br>* typeset	
Additional data availability information:	Tick here if the URLs/accession numbers/DOIs will be available only after acceptance of the manuscript for publication so that we can ensure their inclusion before publication.

Powered by Editorial Manager® and ProduXion Manager® from Aries Systems Corporation

Click here to access/download;Manuscript;MANUSCRIOT.docx

Insecticidal activity of *Ageratina adenophora* (Asteraceae) extract against *Limax maximus* (Mollusca, Limacidae) at different developmental stages and its chemical constituent analysis

Haojun Li [1], Runa Zhao [1], Yingna Pan [1], Hui Tian [1], and Wenlong Chen [1,*]

[1] Provincial Key Laboratory for Agricultural Pest Management of Mountainous Region, Institute of Entomology, Guizhou University, Guiyang 550025, China; frsmncd785@163.com (H.L.); zhao21373@163.com (R.Z.); ynpan1010@163.com (Y.P.); gzdxTH2021@163.com (H.T.)

* Correspondence: wlchen@gzu.edu.cn

Abstract: *Limax maximus*, or great gray slug, is a common ~~pest in agriculture~~agricultural pest. The pest infests crops during their growth phase, creating holes in vegetable leaves, particularly in seedlings and tender leaves. A study was conducted to assess the insecticidal activity of *Ageratina adenophora* extract against these slugs. Factors such as fecundity, growth, hatching rate, offspring survival rate, protective enzyme activity, and detoxifying enzyme activity were examined in slugs exposed to the ~~sublethal concentration (LC_{50}) of the extract~~extract's sublethal concentration (LC50) for two different durations (24 and 48 h). The phytochemical variability of the extracts was also studied. The LC_{50} value of the *A. adenophora* extract against *L. maximus* was ~~found to be~~ 35.9 mg/mL. This extract significantly reduced the hatching rate of eggs and the survival rate of offspring hatched from exposed eggs compared with the control. The lowest rates were observed in those exposed for 48 h. The survival, growth, protective enzyme~~ activity, and detoxification activity of both~~, and detoxification activity of newly hatched and 40-day-old slugs decreased. The *A. adenophora* extract contained tannins, flavonoids, and saponins, ~~which may contribute~~possibly contributing to their biological effects. These results suggest that the extract could be used as an alternative treatment for slug extermination, effectively controlling this species.

Keywords: *Limax maximus, Ageratina adenophora,* molluscide, agricultural pest control, plant extract

1. Introduction

The terrestrial slug *Limax maximus* (Limacidae) is a significant agricultural pest in China owing to its widespread distribution and numerous host species. Both adults and larvae can cause damage to crops, especially those in the Solanaceae, Cruciferae, and Leguminosae families. They leave white mucus traces and sticky feces after crawling, which can harbor bacteria. The damage caused by slugs, including bite marks on leaves, fruits, and stems, significantly impacts the quality of agricultural products and crop safety [1]. Slugs ~~not only harm crops but are also~~harm crops and are closely associated with plant pathogens and the transmission of parasites to humans, livestock, and wild mammals, posing a considerable threat to human health.

Currently, the control of *L. maximus* primarily involves spraying chemical pesticides, such as tetrameric acetaldehyde [2]. However, the prolonged use of slug control agents can lead to slug resistance and diminish

the control efficacy. Additionally, these substances can harm the environment, especially by causing water pollution [3].

Ageratina adenophora (Asteraceae) is a perennial herb of the Compositae family, native to South America [4]. It was introduced into Lincang, Yunnan, China, from Myanmar in the 1940s and has become widely distributed in Yunnan, Guizhou, Sichuan, Guangxi, and Chongqing. In 2003, it was listed as an alien invasive species in China. This plant often thrives in tidal wetlands or on hillside roads, exhibiting robust

fruiting ability and rapid spread. *A. adenophora* has strong vitality and fecundity, displaying broad adaptability [5]. It invades farmlands, gardens, forests, and other habitats, forming dominant communities, suppressing the growth of other plants, and disrupting plant diversity, especially in southwest China [6]. Studies have shown that this plant contains active principles in its chemical constituents, such as flavonoids, saponins, and tannins, possessing biocidal activities, including molluscicidal properties [1, 7-9]. These findings underscore the significance of this plant in controlling mollusk species.

This study aimed to confirm the presence of flavonoids, tannins, and saponins in the extract of *A. adenophora* and to analyze and identify their contents~~ in the extract~~. We calculated the effects of LC_{50} on eggs, larvae, and adult slugs and investigated the impact of 24 and 48 h exposure to semi-lethal concentrations on different life stages of slugs.

2. Materials and Methods

2.1. Slugs and plants

The eggs, newly hatched slugs, and 40-day-old slugs utilized in this study were sourced from the ecological experimental field of Professor Chen at the Institute of Entomology, Guizhou University (latitude: 26°25 ′39.62″N; longitude: 106°40 ′5.81″E; 1090 m altitude). Slugs were reared following a standard method [10]. In summary, *L. maximus* were reared in plastic containers with dimensions of 9 cm in diameter and 6 cm in length, containing 50 g of moist culture soil (120° 1 h), and a mixture of fresh vegetables and protein-rich feed mass (3:1) for feeding. The top of the container was covered with a plastic film with small holes for ventilation. Environmental conditions were maintained at 25 ± 1°C, 70 ± 5% relative humidity, and a 16:8 h (L:D) photoperiod in an environmental chamber.

Leaves of *A. adenophora* were collected from the campuses of Guizhou University (latitude: 26°42 ′69.93″N; longitude: 106°66 ′95.34″E; 1083 m altitude) in Guiyang in December 2022. These leaves were cleaned, dried in an oven at 60°C for 24 h, and ~~then ~~ground into a powder using a mechanical grinder. The plant extract was prepared by infusing 100 g of leaf powder in 1000 mL of distilled water for 72 h, ~~followed by~~then filtering the obtained suspension and testing it with an aqueous solution. The sublethal concentration (LC_{50}) for adult slugs was calculated for subsequent hatching and survival experiments.

2.2. Phytochemical analysis of aqueous extract of *A. adenophora*

To determine the presence of various compounds such as flavonoids, saponins, and tannins, 5.0 g of dried powder was boiled in distilled water for 10 min. The solution was then filtered after cooling, and the supernatant was used for detection. The presence of flavonoids was confirmed using a 10% sodium hydroxide colorimetric analysis [11]. The identification of saponins and the determination of the foaming index in the water extract were performed according to the standard method [12, 13]. To identify tannins in the *A. adenophora* extract, a 2.5% agar solution was prepared in distilled water. Colorimetric tests were performed with a 2% ferric chloride ($FeCl_3$) solution to distinguish between types of tannins (condensed or hydrolyzed).

Condensed tannins were quantified using the Stiasny method [14], which was repeated three times, and the presence of tannins was confirmed based on the turbidity of the water extract.

2.3. Determination of flavonoids, saponins, and tannins in *A. adenophora* aqueous extract

The sample solution was prepared with 1000 mL of the extract. The flavonoid content was assessed using a standard method [15], and rutin was utilized as a standard control. Briefly, 2 mg of rutin was dissolved in ethanol via ultrasonication to create a standard solution of 200 μg mL^{-1}. Volumes of 0, 0.1, 0.2, 0.3, 0.3, 0.4, 0.5, and 0.6 mL were precisely measured and added to 2 mL of water, labeled as 1–7, and 100 μL respectively. The corresponding reagent without rutin served as the blank reference. The absorbance (OD) value was read at 410 nm using a full-wavelength microplate reader, and the standard curve was calculated [16]. For the color operation, 1.0 mL of the sample solution was used, and the OD value was determined at a wavelength of 410 nm using a full-wavelength enzyme labeling instrument. This process was repeated six times, and the data were recorded and analyzed. The saponin content was determined using a modified method [17]. To 20 μL of the aqueous extract of *A. adenophora,* after the evaporation of ethanol, 5 mL of 72% sulfuric acid and 0.5 mL of 8% vanillin ethanol solution were added. The solution was gently mixed, and the OD was determined at 544 nm using a full-wavelength microplate reader. The standard curve of ginsenoside Re was employed to calculate the saponin content. The experiment was repeated six times. The total tannin content was estimated using a previously described procedure [18]. To 1 mL of the extract, 5.0 mL of water, 1.0 mL of sodium tungstate–sodium molybdate mixed solution, and 3.0 mL of sodium carbonate solution were successively added for the color reaction. The OD value was determined at 765 nm using a full-wavelength microplate reader. The standard curve of gallic acid was used for determining the tannin content. The experiment was repeated six times for statistical analysis.

The contents of flavonoids, saponins, and tannins in *A. adenophora* extract were calculated [19] according to the following formula:

$$(\%) = \left(\frac{m \times V_2}{V_1 \times M \times 1000}\right) \times 100\% \quad (1)$$

Where, m is the mass of flavonoids, saponins, and tannins; V_1 is the sampling volume of the sample solution; V_2 is the constant volume of the solution; and M is the sample mass.

2.4. Analysis of hatching rate of exposed eggs

The LC_{50} of the *A. adenophora* aqueous extract was utilized for bioassays against the eggs of great gray slugs. In each Petri plate (diameter 9 cm), 10 1-day-old eggs were placed. The eggs were then sprayed with 20 mL of the extract. For the control group, the eggs were treated with distilled water only. After 24 and 48 h, the eggs were transferred to plastic containers. The hatching of eggs was observed daily for ~~a period of~~ 30 days. Each treatment was replicated four times. The experiment was conducted under ~~conditions of~~ 21°C– 24°C and 84% relative air humidity. The hatching rate (%) was determined using the following formula [20]:
hatching rate (%) = (number of slugs hatched/total number of eggs) × 100.

2.5. Survival rate of newly hatched offspring

The hatched slug larvae were maintained under the previously described conditions. They were provided ~~with~~ a diet comprising fresh vegetable leaves and a protein-rich feed mixture (3:1). The incubator was moistened with distilled water every 2 days. Mortality and maturity assessments were conducted every 3 days over ~~a span of~~ 120 days, and any deceased individuals were promptly removed from the incubator. The survival

rate (%) was calculated using the following formula [21]: Survival rate (%) = (number of unviable embryos/total number of embryos) × 100.

2.6. Effects of LC_{50} on the growth, survival, and reproduction of newly hatched larvae and *L. maximus*

In this experiment, 60 newly hatched slug larvae and 40-day-old slugs were selected, exhibiting an average size of 6.62 ± 0.57 mm (for the newly hatched larvae) and 38.48 ± 2.14 mm (for the 40-day-old slugs). *L. maximus* were organized into groups (10 in each group, with four repetitions) and placed in plastic perforated boxes filled with presterilized culture soil. A precalculated LC_{50} of *A. adenophora* extract (20 mL) was sprayed in each box. The control group, containing the same number of *L. maximus*, was treated with distilled water only and sealed with cotton cloth and elastic rubber to prevent slug escape. At the end of the exposure period, the slugs were transferred to another container and maintained using the method described above.

Growth was assessed by measuring body length using Vernier calipers [22]. The mortality rate was evaluated every 3 days over~~ a period of~~ 120 days, and any deceased individuals were promptly removed from the incubator via direct observation of *L. maximus*. Sexual maturation was also assessed at the same frequency, determined by the presence of eggs in the womb [23].

2.7. Effects of LC_{50} on the activities of protective and detoxifying enzymes in newly hatched larvae and slugs

Superoxide dismutase (SOD), peroxidase (POD), and catalase (CAT) constitute the protective enzyme system in mollusks. These enzymes work ~~in conjunction~~together to maintain intracellular free radicals at a low level, effectively preventing free radical toxicity. On the contrary, cytochrome P450 (CYP450), glutathione-Stransferase (GST), and acetylcholinesterase (AChE) are prominent detoxification enzymes in mollusks. These enzymes ~~play an important role~~are important in breaking down exogenous toxins and maintaining normal physiological metabolism [24]. The average sizes of the newly hatched slug larvae and 40-day-old slugs were ~~measured to be ~~6.62 ± 0.57 mm and 38.48 ± 2.14 mm, respectively. The slugs were placed in plastic boxes with holes and raised in the same environment ~~as ~~mentioned earlier. Healthy slugs were subjected to the precalculated LC_{50}. The control group, comprising the same number of slugs, was treated with distilled water only. The treatment duration was set at 24 and 48 h, and each ~~treatment ~~was repeated five times within these periods. After the treatment, the slugs were preserved in liquid nitrogen and weighed postmortem. A 10% adult homogenate was prepared by adding a volume of homogenate medium (0.1 mol/L saline, pH 7–7.4) nine times the ~~weight (in grams) of the sample~~sample's weight (in grams) under an ice bath. The mixture was then centrifuged at 4000 rpm for 10 min. The supernatant was collected, and its OD value was determined at 420 nm with a 1 cm light path using a 100 µL sample.

Inhibition activities of protective enzymes, i.e., SOD, POD, and CAT, and detoxification enzymes, i.e., CYP450, GST, and AChE, were determined as per the commercial enzyme-linked immunosorbent assay (ELISA) kit (Suzhou Mengxi Biopharmaceutical Technology Co., Ltd.). The newly hatched slug larvae and adults treated with LC_{50} of *A. adenophora* aqueous extract or distilled water for 24 and 48 h were collected for enzyme assay. The treated slugs were stored at −80°C.

2.8. Statistical analysis

The regression equations for the standard samples used to determine flavonoids, saponins, and tannins were calculated and analyzed using the Microsoft Excel 2021 software. The LC_{50} value was computed using the probit analysis in IBM SPSS Statistics 25 software [9]. The concentration–response curve was established using the same test [25]. To compare hatchability, mortality, growth, and development of *L. maximus* exposed to LC_{50}, the Kruskal–Wallis ~~method and the Student–Kewman–Keuls method~~and the Student–Kewman–Keuls

methods ($p < 0.05$) were employed. The hatching rate, mortality, and growth of slugs exposed to LC_{50} were compared using the BioEstat 5.0 software. Statistical analysis of protective ~~enzyme~~ and detoxification enzyme activity data was performed using IBM SPSS Statistics 25. The difference between treatments was analyzed using single-factor analysis of variance, and the significant difference was tested using the least significant difference method. Curves were plotted using the Origin 2021 software.

3. Results

3.1. Phytochemical analysis of *A. adenophora* extract

After adding the sodium hydroxide solution, ~~the color of~~ the *A. adenophora* extract changed from light yellow to red, a characteristic color associated with chalcone and gold ketone, thus confirming the presence of flavonoids in the aqueous extract. Tannin presence was confirmed using the agar solution test, with the emergence of a moss green color affirming the presence of condensed tannin upon ~~the addition of~~adding ferric chloride. This finding was further validated using the Stiasny test. The results of these analyses unequivocally indicated the presence of saponins, as evidenced by a foam index of 100.

3.2. Determination of flavonoids, saponins, and tannins in the extract

3.2.1. Flavonoid content

The regression equation for the rutin reference substance was expressed as $y = 0.1744x + 0.0007$, with an R^2 value of 0.9946. This signifies a robust linear relationship for the rutin reference substance within the specified range of 0.00-0.10 μg. Figure 1 represents the standard curve, plotting mass (μg) on the X-axis and OD values on the Y axis.

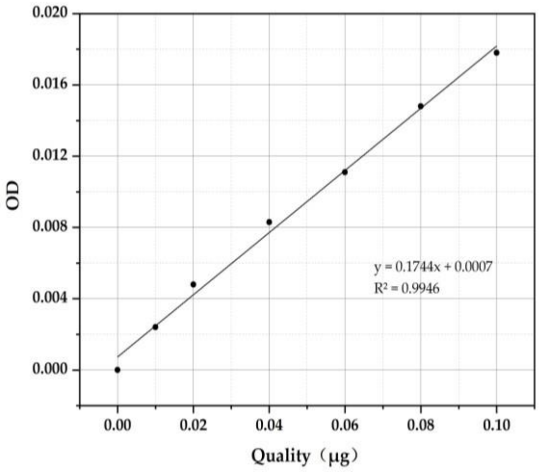

Figure 1: Rutin standard curve

After conducting six repeated tests, the content of flavonoids in the *A. adenophora* extract ranged from 4.72% to 5.02%, as indicated in Table 1.

Table 1. Determination of flavonoids in *Ageratina adenophora* extract after six times of detection

Serial number	OD	Flavonoid content	Average content	Standard deviation	RSD

		(%)	(%)		(%)
1	0.287	5.02			
2	0.267	4.72 4.91			
3	0.277	4.83			
4	0.273		4.87	0.1119	2.30
5	0.282	4.96			
6	0.271	4.79			

3.2.2. Saponin content

The regression equation for the ginsenoside Re reference substance was described as y = 0.0427x + 0.0119, with an R^2 value of 0.9965. This indicates a strong linear relationship for the ginsenoside Re reference substance within the specified range of 0.00–10.00 μg. Figure 2 represents the standard curve, plotting mass (μg) on the X-axis and OD values on the Y axis.

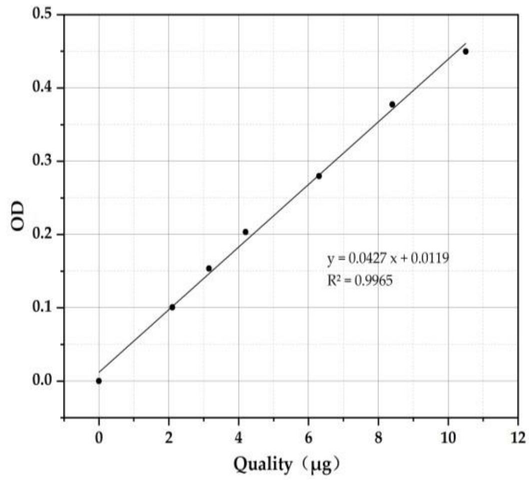

Figure 2: Ginsenoside Re standard curve

Following six repeated tests, the content of saponins in the *A. adenophor*a extract ranged from 1.79% to 1.89%, as shown in Table 2.

Table 2. Determination of saponins in *Ageratina adenophora* extract after six times of detection

**Serial number**	**OD**	**Saponin content** (%)	**Average content** (%)	**Standard deviation**	**RSD** (%)
1	0.159	1.87			

2	0.141	1.79	1.85	0.041	2.21
3	0.162	1.88			
4	0.149	1.83			
5	0.145	1.81			
6	0.165	1.89			

3.2.3. Tannin content

The regression equation for the gallic acid reference substance was $y = 0.2082x + 0.0038$, with an R^2 value of 0.9968. This signifies a good linear relationship for the gallic acid reference substance within the specified range of 0.00–0.60 μg. Figure 3 represents the standard curve, plotting mass (in μg) on the X-axis and OD values on the Y axis.

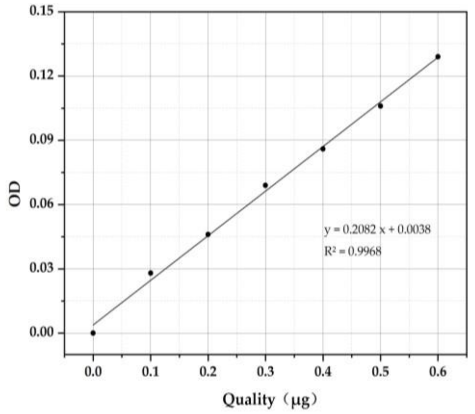

Figure 3: Gallic acid standard curve

After conducting six repeated tests, the content of tannins in the *A. adenophora* extract ranged from 1.31% to 1.40%, as shown in Table 3.

Table 3. Determination of tannins in *Ageratina adenophora* extract

**Serial Number**	**OD**	**Tannin Content** （%）	**Average Content** （%）	**Standard Deviation**	**RSD** （%）
1	0.049	1.40			
2	0.046	1.34			
3	0.045	1.32			
4	0.044	1.31	1.35	0.0348	2.57
5	0.047	1.36			

6	0.048	1.38

3.3. Calculation of LC_{50} of *A. adenophora* aqueous extract

Using statistical data from IBM SPSS Statistics 25 software and Probit for analysis to calculate LC_{50}, the determined LC_{50} was 35.9 mg/mL. The ratio of concentration to response is shown in Figure 4.

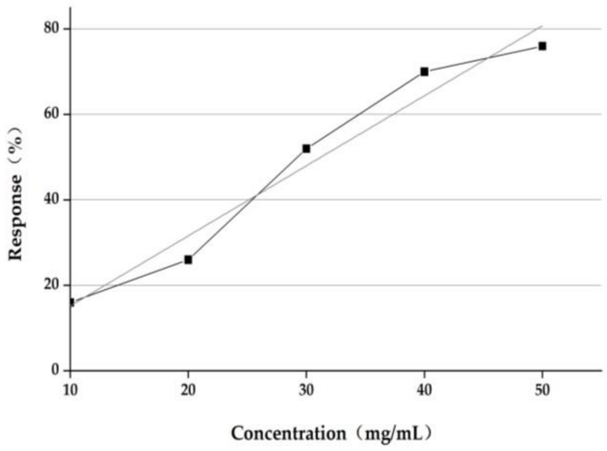

Figure 4: Concentration–response ratio of *Limax maximus* to *Ageratina adenophora* extract. Assessment response = fatality rate, expressed as a percentage of deceased *L. maximus*.

3.4. Analysis of hatching rate after 24 and 48 h of egg exposure

The extract of *A. adenophora* was diluted to five gradient concentrations, and 50 slugs were selected for each concentration. Subsequently, the slugs were sprayed with the extract. After 7 days, the number of slug fatalities was recorded. The results of the toxicological test are presented in Table 4. The results demonstrated that the *A. adenophora* extract ~~had adverse effects on~~adversely affected the hatching rate of eggs exposed for 24 h (H = 11.3742; p = 0.0018) and 48 h (H = 20.7134; p = 0.0002). Moreover, it was observed that as the exposure time increased, the hatching rate decreased (H = 4.127; p = 0.0319). However, there was no significant difference in the average hatching value of the control group between the two periods (H = 0.0556; p = 0.5319). Specifically, the hatching rates after 24 and 48 h of exposure were 97.9% and 94.2%, respectively (Table 5).

Table 4. Determination of toxicity of *Ageratina adenophora* extract to adult slugs

Reagent concentration (mg/mL)	Slug number	Number of deaths
10	50	38
20	50	35
30	50	26
40	50	13
50	50	8

Table 5: Hatching rate of *Ageratina adenophora* aqueous extract observed after 24 and 48 h of exposure. (Average value, standard deviation, range of variation, and hatchability percentage). [a,b,c] = means followed by distinct letters indicate significant differences as per the Kruskal–Wallis test ($p < 0.05$).

Groups		Hatchability X ± SD	Range of variation	Hatchability Percentage (%)
Control	24 h	39.16 ± 0.41^a	(38–40)	97.9
	48 h	37.68 ± 1.09^a	(36–39)	94.2
Exposed	24 h	22.64 ± 3.10^b	(19–26)	56.6
	48 h	7.48 ± 1.65^c	(5–9)	18.7

3.5. Survival rate of offspring hatched from exposed eggs

In comparison with the control group, the survival rate of hatched offspring from eggs exposed to the extract experienced a significant decrease (24 h: $H = 9.9698$, $p = 0.0037$; 48 h: $H = 12.9856$, $p = 0.0031$) (Figure 5). Furthermore, the survival rate of hatched offspring from eggs exposed for 24 h was higher than that for 48 h ($H = 7.5397$; $p = 0.0036$). All slugs exposed to the extract for 48 h had perished by the 87th day of observation. There was no significant difference in the survival rate of hatched offspring from eggs in the control group between the two ~~time~~ periods (24 h: 90.3%, 48 h: 87.8%; $H = 7.4856$, $p = 0.2468$) (Figure 5). Throughout the observation period, neither the slugs in the exposure group nor those in the control group displayed sexual maturity.

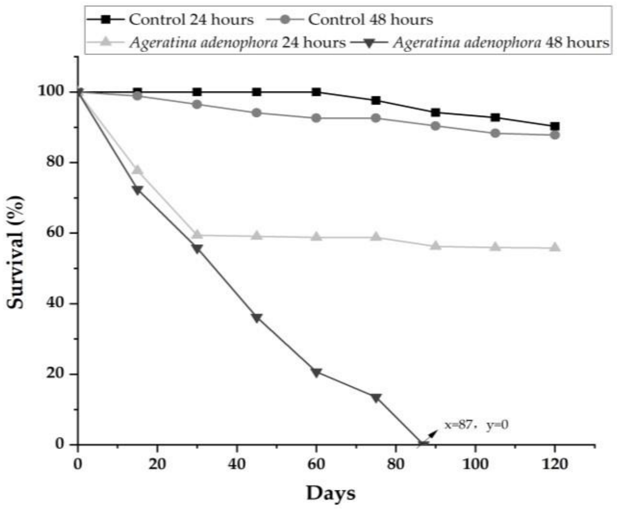

Figure 5: The survival rate of newly hatched offspring from eggs exposed to *Ageratina adenophora* extract for 24 and 48 h over ~~a 120-day period~~120 days

3.6. Effects of LC_{50} on the growth, survival, and reproduction of newly hatched *L. maximus* larvae exposed for 24 and 48 h

Growth analysis of the exposed group revealed that at 60 days, there was no significant difference in the average size between the 24 h exposure group and the control group (H = 0.5677; p = 0.4772). However, the exposure time ~~demonstrated an effect on~~affected the growth rate, indicating that the longer the exposure time, the slower the growth rate. The average size of the 48 h exposure group was lower than that of the control group (H = 8.6414; p = 0.0036). Specifically, the ~~average size at 24 and 48 h in the control group~~control group's average size at 24 and 48 h was 52.21 ± 3.94 mm and 47.66 ± 2.48 mm, respectively. In contrast, the average size ~~at 24 and 48 h in the exposed group~~in the exposed group at 24 and 48 h was 51.06 ± 3.13 mm and 36.75 ± 2.24 mm, respectively.

The survival rate of newly hatched slug larvae exposed to *A. adenophora* extract was notably lower than that of the control group (24 h: H = 5.7587; p = 0.022148h; H = 5.9039; p = 0.0221). Furthermore, a correlation was observed between the exposure time and the survival rate of slug larvae, indicating that the longer the exposure, the lower the survival rate (H = 5.0128; p = 0.0453). However, the ~~duration of exposure did not have a significant effect on~~exposure duration did not significantly affect the survival rate of the control group (H = 0.29; p = 0.5273). All slugs in the 24 h exposure group succumbed at the end of 108 days, and those in the 48 h exposure group perished by the end of 81 days. The results are displayed in Figure 6.

At the end of the 75-day observation, the normal maturation time of *L. maximus* was consistent with the normal maturation time of the slugs. Only 28% of slugs survived in the 24 h exposure group to reach sexual maturity at approximately 68 ± 7.1 days. In comparison, the control group exhibited an average maturation time of 55 ± 4.5 days during the same period. In the 48 h exposure group, a mere 11% of slugs survived to reach sexual maturity, with an average maturation time of 73 ± 1.8 days, whereas the control group's average maturation time was 58 ± 4.9 days.

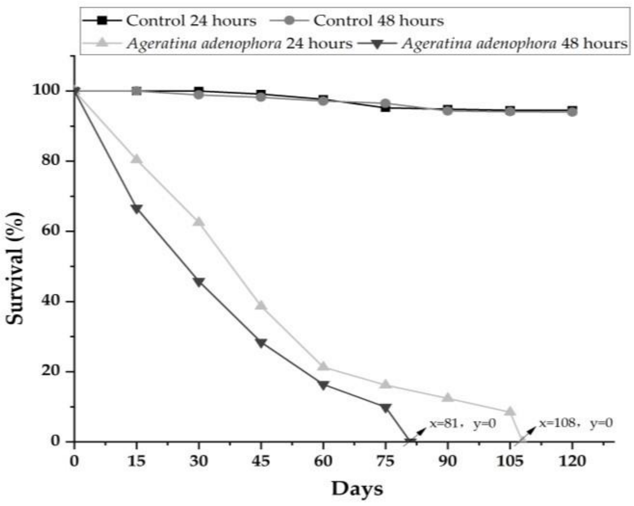

Figure 6: Observation of newly hatched slug larvae exposed to *Ageratina adenophora* extract for 24 and 48 h over a period of 120 days.

3.7. Effects of LC_{50} on the growth, survival, and reproduction of 40-day-old *L. maximus*

Growth analysis of the exposed group revealed a higher mortality rate compared with the control group at 30 days. A significant difference in the average size of slugs between the exposed group and the control group was observed (24 h: H = 11.0795 ; p = 0.0004 ; 48h: H = 14.8655 ; $p < 0.0001$). Specifically, the average sizes of the 24 and 48 h exposure groups were 44.96 ± 2.57 mm and 40.18 ± 2.34 mm, respectively, whereas those of the control group were 51.74 ± 3.05 mm and 50.83 ± 2.99 mm, respectively.

In the exposed group, *L. maximus* did not experience mortality on the first day, and the initial death occurred in the 48 h exposure group on the second day. All slugs in the 24 h exposure group perished at the end of 117 days, and all those in the 48 h exposure group succumbed at the end of 99 days. The survival rate of the exposure group was significantly lower than that of the control group (24 h: H = 5.4988; p = 0.0212; 48 h: H = 5.8271; p = 0.0351). Conversely, there was no significant difference in the survival rate between the two time periods in the control group (H = 0.0286; p = 0.9463.) Notably, the survival rate of slugs exposed to the extract for 48 h was higher in the first 3 weeks, then decreased gradually, and experienced a rapid decline from 30 to 75 days. These results indicate the presence of *A. adenophora* extract in *L. maximus*. The survival rate of slugs decreased linearly in the 24 h exposure group. The results are depicted in Figure 7. In the 24 h exposure group, only 19% of the surviving slugs reached sexual maturity, with an approximate maturity period of 64 ± 5.4 days. No sexual maturity was observed in the surviving slugs in the 48 h exposure group. In contrast, the control group exhibited sexual maturity rates of 54% and 46.2% for the 24 and 48 h exposures, respectively, with average maturation times of 53 ± 3.8 days and 55 ± 2.4 days, respectively.

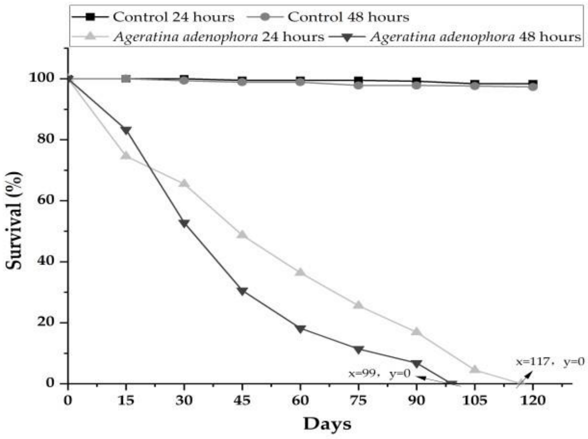

Figure 7: Survival rate of 40-day-old *Limax maximus* exposed to *Ageratina adenophora* extract for 24 and 48 h, observed over ~~a 120 day period~~120 days.

3.8. Effects of LC_{50} on the activities of protective and detoxifying enzymes in newly hatched larvae and 40day-old slugs

The effects of LC_{50} on the activities of protective enzymes in slugs treated with *A. adenophora* extract for 24 and 48 h were significantly lower than those of the control. This finding suggests that *A. adenophora* extract ~~has the ability to~~can inhibit the activities of SOD, POD, and CAT enzymes. Specifically, after a 24 h treatment with *A. adenophora* extract, the activities of protective enzymes (SOD, POD, and CAT) in adult slugs decreased significantly, indicating the inhibitory effect of *A. adenophora* extract on the protective enzymes in adult slugs. This trend continued when the adult slugs were treated with *A. adenophora* extract for

48 h. The results demonstrates that the activities of protective enzymes in slugs were impacted upon treatment with *A. adenophora* extract.

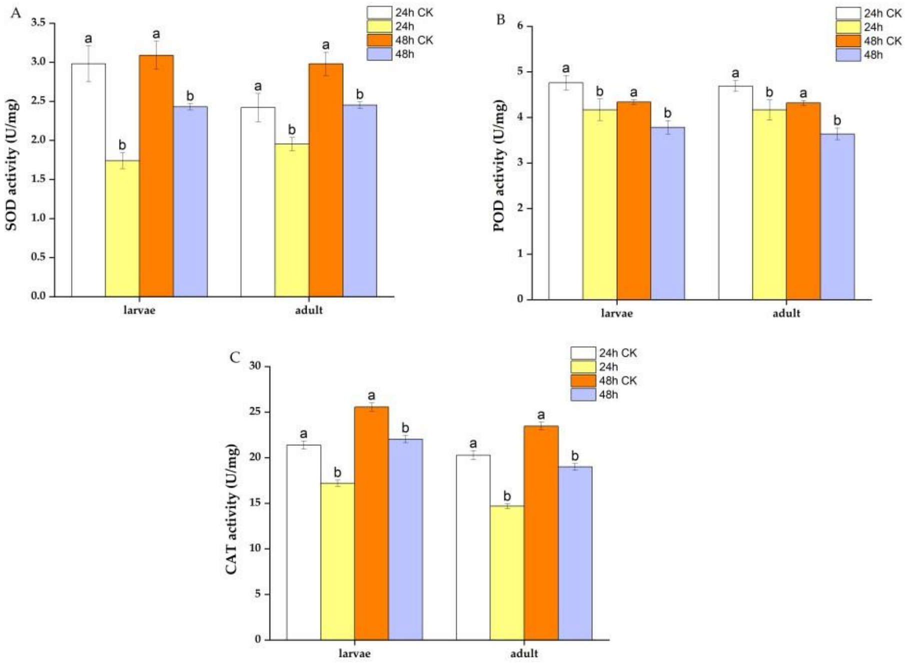

Figure 8: Protective enzyme activities of slug larvae and adults treated with LC_{50} for 24 and 48 h, respectively. The letters above the columns denote the results of multiple comparative experiments performed using Tukey tests on the enzyme activities of slug larvae and adults after LC_{50} treatment. Different lowercase letters indicate statistically significant differences.

The LC_{50} effect on the detoxifying enzyme activity in slugs treated with *A. adenophora* extract was observed over ~~periods of 24 and 48 h~~24 and 48 h periods. The activity of the detoxifying enzyme AChE in both slug larvae and adults was significantly higher than that in the control group, indicating a significant activation of AChE in these groups. However, after being treated with *A. adenophora* extract for the same durations, the activities of the detoxification enzymes CYP450 and GST in slugs were significantly lower than those in the control group. This result suggests that the CYP450 and GST enzymes in ~~both~~ adult and larval slugs were inhibited significantly following treatment with *A. adenophora* extract.

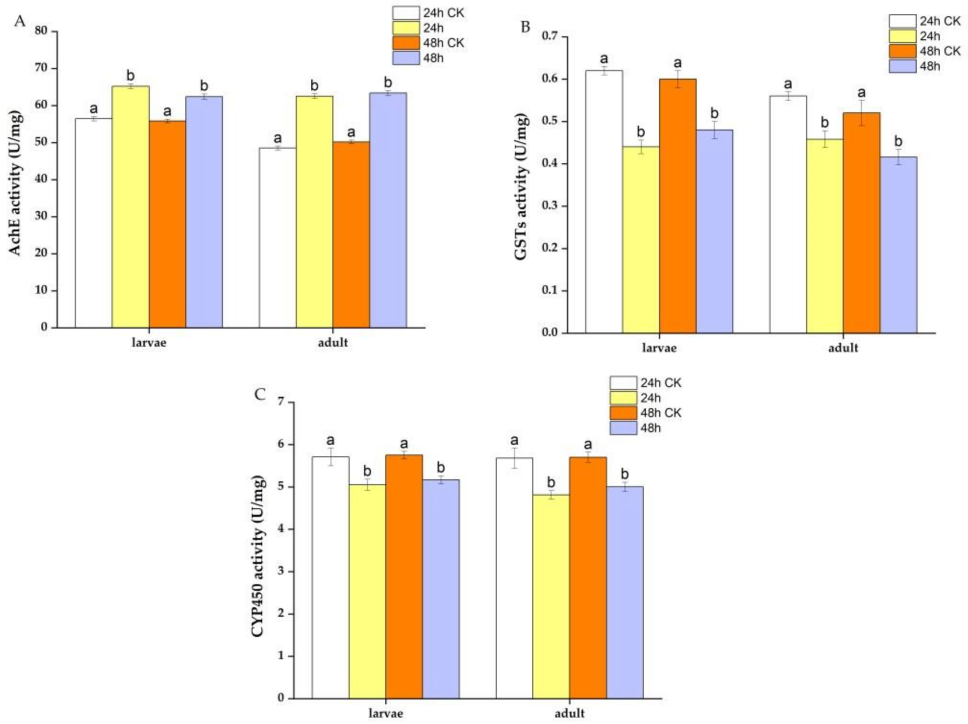

Figure 9: Detoxifying enzyme activities of slug larvae and adults treated with LC_{50} for 24 and 48 h, respectively. The letters above the columns denote the results of multiple comparative experiments performed using Tukey tests on enzyme activities of slug larvae and adults after LC_{50} treatment. Different lowercase letters indicate statistically significant differences.

4. Discussion

In this study, we observed that *A. adenophora* aqueous extracts showed insecticidal activity against slugs at various life stages. The insecticidal effects were attributed to ~~the presence of~~ flavonoids, saponins, and tannins, which are secondary metabolites found in the extract. However, it is important to note that ~~there may be other undetected secondary metabolites with~~other undetected secondary metabolites may have potential bactericidal activity, and those with low content might still exert weak germicidal activity. Hence, further research is required to investigate the germicidal activity of these metabolites.

The *A. adenophora* extract influenced the hatching rate of *L. maximus*, indicating that the molecular structure of the active plant ingredient facilitates penetration into the embryo-associated membrane pores. However, prior research has reported that the high molecular weight of the egg membrane can impede drug penetration [26]. Notably, the mortality rate of eggs exposed to the extract for 48 h surpassed that of the 24 h exposure, signifying a more pronounced effect on the hatching rate of slug eggs with longer exposure times.

Temperature is a potential factor affecting the egg hatching rate ~~as well as~~and slug growth and development [27]. Extremes in temperature, whether high or low, can reduce mollusk vitality, leading to states of lethargy when environmental conditions are unfavorable. Additionally, high temperatures elevate mollusk oxygen consumption, accelerating the ~~rate of energy depletion~~energy depletion rate within the organism [28]. In this study, the temperature was controlled within the ~~range of~~ 21°C–24°C.

Souza et al. (1992) confirmed the high sensitivity of newly hatched snails to cashew nut shell extracts [29]. In a related study, Gohar et al. (2014) reported molluscicidal activity against newly hatched *Biomphalaria alexandrina* using *Callistemon viminalis* (Sol. ex Gaertner) G.Don ex Loudon [30]. However, our investigation demonstrated significant changes in adult *L. maximus* exposed for 24 and 48 h after 45 days,

including reduced food intake, retarded movement, a darker body surface color, and somnolence. These alterations led to differences in growth, development, and sexual maturity compared with the control group [31].

Under stress conditions, insects accumulate superoxide anion radicals, hydroxyl radicals, hydrogen peroxide, and other reactive oxygen species, resulting in organismal damage. However, insects possess protective enzymes, including SOD, POD, and CAT, which scavenge excess free radicals [32, 33]. These enzymes work synergistically to maintain the dynamic balance of free radical metabolism to protect the insect from injury or minimize its impact [34]. Research has indicated that *Nilaparvata lugens* and *Sogatella furcifera* significantly increased the activities of SOD, POD, and CAT in rice plants infected with rice blackstreaked dwarf disease or southern rice black-streaked dwarf disease [35]. Our results implied that the LC_{50} of *A. adenophora* extract had an inhibitory effect on SOD activity in slugs, inhibition–activation–inhibition effect on POD activity, and activation–inhibition effect on CAT activity.

CYP450, GST, and AChE play important roles in breaking down exogenous toxicants and maintaining normal physiological metabolism [36]. ~~Of these, GST catalyzes sulfhydryl coupling between electrophilic groups of toxic substances and reduced~~GST catalyzes sulfhydryl coupling between electrophilic groups of toxic substances and reduces glutathione, enhancing their hydrophobicity for easy excretion from the body [37]. Methylvitamin salt significantly inhibited AChE activity in the 2nd instar larvae of *Lymantria dispar*, and GST activity was activated after 24 h of treatment [38]. Carbendazim can inhibit AChE completely in earthworms and activate GST completely. It can induce the activation of CYP450 at low concentrations and inhibit it at high concentrations [39]. Under the sublethal concentration stress of tetrameric acetaldehyde, the activity of AChE increased initially~~ and then~~. Then it decreased in the gill and abdominal foot of *Pomacea canaliculata*, whereas the activity in the liver and intestine decreased initially and then increased [40]. Our results demonstrated that after treatment with the sublethal concentration of *A. adenophora* (LC_{50}), the activities of CYP450 and GST in slugs were inhibited to varying degrees, but the activity of AChE increased. ~~Owing to the high content of flavonoids in the extrac~~Due to the extract's high flavonoid content, AChE was released in slugs, leading to dehydration and death.

In this study, the LC_{50} extract of *A. adenophora* ~~was found to contain the highest content of flavonoids~~contained the highest flavonoid content, averaging 4.87%. This was followed by saponins and tannins, which were ~~present ~~at 1.85% and 1.35% respectively. It can be inferred that flavonoids played a significant role in the extract, although it cannot be denied that compounds ~~present ~~in smaller quantities also exerted an effect. Future studies ~~are needed to~~must confirm whether separately extracted flavonoids from *A. adenophora* extract will have a more significant lethal effect on slugs.

Flavonoids may inhibit the detoxification system of snails. Some studies have observed changes in the CYP450 enzyme of the land snail *Cantareus aspersus* after exposure to tobacco leaves [41, 42]. ~~In this study, a decrease in CYP450 enzyme activity was noted~~This study noted a decrease in CYP450 enzyme activity after *L. maximus* came into contact with *A. adenophora* extract. This enzyme is part of a protein family involved in detoxification and can degrade various foreign substances [43]. Additionally, a study has shown that flavonoids can activate AChE, leading to a huge release of AChE in slugs [44]. This release destroys the special mucus produced by slugs, causing rapid dehydration, destruction of body surface cells, and death due to significant body fluid loss [45]. These findings are aligned with the results of AChE enzyme activity determined in this study, supporting the idea that flavonoids can activate AChE activity *in vivo*. Previous studies have concluded that saponins exhibit hemolytic toxicity, which can destroy red blood cell membranes and cause cytoplasmic extravasation, leading to red blood cell disintegration [46, 47]. Tannin is a ~~type of ~~polyphenol containing several hydroxyl groups; it can complex with protein and cause precipitation, thereby inactivating it [48, 49]. However, th~~e specific effects of saponins and tannins on slugs were not confirmed in this study~~is study did not

confirm the specific effects of saponins and tannins on slugs. Further research on the specific effects of these metabolites on slugs is an important future direction.

Exposure to toxic substances or gases can cause physiological stress in slugs, re~~sulting in the reduction of~~ducing carbohydrate and glycogen reserves [50]. This triggers the depletion of the body's protein as an alternative energy source [51]. Studies have shown that mollusks exposed to toxins exhibit increased levels of hemolymph protein and uric acid, indicating protein degradation for energy [50]. However, proteins may precipitate after complexation and cannot be used as an energy substance [52], resulting in an energy imbalance and ultimately death in the slug.

Currently, chemical agents are predominantly used for mollusk control in Chinese agriculture [3]. However, the drawbacks of chemical control are becoming increasingly apparent. ~~For more effective control of these species, plant extracts should be effective against all life stages of the target organism~~Plant extracts should be effective against all life stages of the target organism for more effective control of these species. Considerations for agricultural application include factory production; extract preparation and treatment; selectivity at low concentrations; and potential harm to humans, the environment, and crops. Comprehensive studies on plant secondary metabolites in terrestrial slug species should be strengthened. This study confirmed that *A. adenophora* extract has a lethal effect on slugs, but it is unclear which secondary metabolites have the greatest killing activity. Future research should focus on a specific class of secondary metabolites or combinations ~~thereof~~ to maximize the lethal effect. Moreover, field experiments are necessary for practical farmland control applications.

5. Conclusions

A. adenophora is an invasive plant characterized by its wide distribution and strong fecundity. The aqueous extract of *A. adenophora,* which contains flavonoids, saponins, and tannins, has been shown to affect the activity of *L. maximus* at all stages of the yellow slug's life cycle. This extract inhibits egg hatching and induces morphological changes in eggs. Interestingly, adults appear ~~to be~~ more sensitive to the extract's toxicity than larvae. In addition to significant body fluid loss, behavioral changes, such as lethargy and decreased vitality, were observed in adult slugs. These findings suggest that the extract disrupts the balance within *L. maximus*. Therefore, the aqueous extract of *A. adenophora* contains active ingredients that can kill slugs. Given its water solubility, the extraction process ~~is likely to~~will likely be environmentally friendly. Furthermore, it holds promise ~~as a means of effectively controlling the biological invasion of *A. adenophora* and slug species that pose a threat to~~to effectively control the biological invasion of A. adenophora and slug species that threaten other agriculturally important crops.

Author Contributions: Conceptualization, H.L. and W.C.; methodology, H.L. and H.T.; formal analysis, R.Z. and Y.P.; investigation, H.L. and Y.P.; writing—original draft preparation, H.L.; validation, R.Z.; writing—review and editing, R.Z. and W.C. All authors have read and agreed to the published version of the manuscript.

Funding: This research received no external funding.

Institutional Review Board Statement: Not applicable

Informed Consent Statement: Not applicable

Data Availability Statement: The data represented in this study are available on request from the corresponding author.

Conflicts of Interest: The authors declare no conflict of interest.

Acknowledgements: The authors wish to thank the Institute of Entomology of Guizhou University for assistance in this research.

References

1. Jiang L, Zhao RN, Tian H, Wu XS, Guo F, Chen WL (2021) Functional Response and Predation Potential of Carabus elysii Adults against the Terrestrial Slug Agriolimax agrestis. Insects 12 (12). https://doi.org/10.3390/insects12121135. https://www.ncbi.nlm.nih.gov/pubmed/34940223
2. Gasparotto Jr, Arquimedes, Mislaine AB, Izabel CP, Diógenes Aparício GC, Celso VN, Benedito Prado DF, Edson R F, Antonio GF (2005) Estudo fitoquímico e avaliação da atividade moluscicida do Calophyllum brasiliense Camb (Clusiaceae). Química Nova 28 (4): 575578. https://doi.org/10.1590/s010040422005000400003.
3. Yu YW, Liu CG, Han YD (2017) Research Progress of slug Control in China. Liaoning agricultural science No.295 (03): 6266. https://kns.cnki.net/kcms/detail/21.1111.S.20170704.0836.032.html
4. Yan J (2016) Color Atlas of Alien invasive plants in China, ShangHai.
5. Yang C (2020) The species, harm, Control status and Future Development trend of Alien species invasion in Guizhou. Agricultural disaster research 10 (04): 144148+150. https://doi.org/10.19383/j.cnki.nyzhyj.2020.04.059.
6. Guizhou entryexit Inspection and Quarantine Bureau of the people's Republic of China, Chinese Academy of Inspection and Quarantine, Agriculture Bureau of Anshun City, Guizhou University. (2012) Quarantine and identification method of Eupatorium adenophorum. General Administration of quality Supervision, Inspection and Quarantine of the people's Republic of China.
7. Vorontsova YA, Yurlova NI, Vodyanitskaya SN, Glupov VV (2010) Activity of detoxifying and antioxidant enzymes in the pond snail Lymnaea stagnalis (Gastropoda: Pulmonata) during invasion by trematode cercariae. Journal of Evolutionary Biochemistry and Physiology 46 (1): 2834. https://doi.org/10.1134/s0022093010010032
8. Marston A, Msonthi JD, Hostettmann K (1984) Naphthoquinones of Diospyros usambarensis; their Molluscicidal and Fungicidal Activities1. Planta medica 50 (3): 27980. https://doi.org/10.1055/s2007969704.
9. Kloos, H, McCullough FS (1985) Ethiopian plants with proven and suspected molluscicidal activity: a new approach in plant evaluation. The Journal of tropical medicine and hygiene 88 (3): 18996. <Go to ISI>://MEDLINE:4068075..
10. Jordaens K, Van Houtte N, Helsen P, Breugelmans K, Jaksons P, Backeljau T (2013) Mixed breeding system in the hermaphroditic land slug Arion intermedius (Stylommatophora, Arionidae). Hereditas 150 (46): 4552. https://doi.org/10.1111/j.16015223.2013.02272.x
11. Mega TP, Santos PD, SouzaMachado A, Ldbc N, Cruz AA (2011) Use of medicinal herbs by patients with severe asthma managed at a Referral Center. Brazilian Journal of Pharmaceutical Sciences 47 (3): 643649. https://doi.org/10.1590/s198482502011000300024.
12. Olefir YV, Sakanyan EI, Tereshina NS, Lyakina MN, Shishova LI (2018) Plant juices and related medicinal preparations: issues of standardization and quality control. Pharmaceutical Chemistry Journal 52 (4): 339342. https://doi.org/10.1007/s1109401818181.
13. Machado AP, Hochman B, Tacani PM, Liebano RE, Ferreira LM (2011) Medical devices registration by ANVISA (Agencia Nacional de Vigilancia Sanitaria). Clinics 66 (6): 10951096. https://doi.org/10.1590/s180759322011000600029.
14. Rodrigues AB, AlmeidaApolonio AA, Alfredo TM, Dantas FGD, Campos JF, Cardoso CAL, Souza KD, De Oliveira KMP (2019) Chemical Composition, Antimicrobial Activity, and Antioxidant Activity of Ocotea minarum (Nees & Mart.) Mez. Oxidative Medicine and Cellular Longevity 2019. https://doi.org/10.1155/2019/5736919.
15. Wang YJ, Zhang C, Ma YL (2017) Determination of total flavonoids in cotyledons of Euphorbia angustifolia and optimization of orthogonal test. Journal of Southwest Medical University 40 (04): 374378. https://kns.cnki.net/kcms/detail/51.1264.R.20170830.1331.026.html.
16. Gao Q, Liu FY, Shi YR, Li JD, Zou WS, Jin N, Cheng CZ (2019) A method based on enzyme labeling instrument to detect the content of polysaccharides in natural plant compound polysaccharides. Journal of Animal Nutrition 31 (02): 922929. https://kns.cnki.net/kcms/detail/11.5461.S.20181229.1012.036.html.

17. Xu FF, Feng HL, Li L, Chu SJ, Yu J, Lou ZH, Cao ZQ (2021) Determination of Total saponins in Panax Notoginseng. Ginseng research 33 (05): 1518. https://doi.org/10.19403/j.cnki.16711521.2021.05.005.
18. Li ZL, Kang X, Li Z, Jia YQ, Chen WQ, Chan XF, Xiao ZG (2019) Determination of tannin in Yuanbao Maple Leaf. Food research and development 40 (09): 177180.
19. Pan J, Shang J, Huang SX (2012) Determination of tannin in feed by sodium tungstatephosphomolybdic acid colorimetric spectrophotometry. Feed industry 33 (07): 5155.
20. Charles S, Virginie D, Didier A, Rachel B, Denise B, Karel DS, Sandra FG, John WG, Henrik H, Thomas HH, Daniel F, Filipe L, Peter M, Leif N, Joerg O, Evelyn RZ, Anne SF, Matthias T, JeanPierre T, Marysia TK, Lennart W, Laurent L (2016) Optimizing the design of a reproduction toxicity test with the pond snail Lymnaea stagnalis. Regulatory Toxicology and Pharmacology 81: 4756. https://doi.org/10.1016/j.yrtph.2016.07.012.
21. Ducrot V, Clare A, Didier A, Denise B, Rebecca B, Sandrine C, Maira C, Marc C, MarieLaure DM, Carole FD, Henrik H, Thomas H, Arne J, Karin LK, Cedric L, Gareth LP, Peter M, Joerg O, Lynsey R, Edward R, Katharina R, Jessica ED, Clemence V, Lennart W, Ruth W, Laurent L (2014) Development and validation of an OECD reproductive toxicity test guideline with the pond snail Lymnaea stagnalis (Mollusca, Gastropoda). Regulatory Toxicology and Pharmacology 70 (3): 605614. https://doi.org/10.1016/j.yrtph.2014.09.004.
22. De S, Bruna A, Alan RTM, Nathália de OM, Flávia Maria Pereira da C, José Dias de SF, Lúcia Pinheiro SP (2022) Chemical Profiling of Strongylodon macrobotrys Flowers and its Molluscicidal Activity. Revista Brasileira de Farmacognosia 31 (6): 848853. https://doi.org/10.1007/s43450021002079.
23. D'ávila S, Elisabeth AB (2005) Influência do substrato sobre o crescimento de Subulina octona (Brugüière) (Mollusca, Subulinidae), sob condições de laboratório. Revista Brasileira de Zoologia 22 (1): 205211. https://doi.org/10.1590/s010181752005000100025.
24. Katusic ZS, Livius Vd'U, Tongrong H (2023) Emerging Roles of Endothelial Nitric Oxide in Preservation of Cognitive Health. Stroke 54 (3): 686696. https://doi.org/10.1161/strokeaha.122.041444.
25. Moellenhoff K, Kirsten S, Franziska K (2022) Identifying alert concentrations using a modelbased bootstrap approach. Biometrics. https://doi.org/10.1111/biom.13799.
26. Giglio ML, Ituarte S, Pasquevich MY, Heras H (2016) The eggs of the apple snail Pomacea maculata are defended by indigestible polysaccharides and toxic proteins. Canadian Journal of Zoology 94 (11): 777785. https://doi.org/10.1139/cjz20160049.
27. Seuffert ME, Silvana B, Pablo RM (2010) Influence of water temperature on the activity of the freshwater snail Pomacea canaliculata (Caenogastropoda: Ampullariidae) at its southernmost limit (Southern Pampas, Argentina). Journal of Thermal Biology 35 (2): 7784. https://doi.org/10.1016/j.jtherbio.2009.11.003.
28. Magura IS, Prevarskaya NB, Zachar J (1985) Temperaturedependent properties of delayed outward current channels in somatic membrane of snail neurones. General physiology and biophysics 4 (1): 979. <Go to ISI>://MEDLINE:4029594.
29. De Souza CP, Mendes NM, JannottiPassos LK, Pereira JP (1992) The use of the shell of the cashew nut, Anacardium occidentale, as an alternative molluscacide. Revista do Instituto de Medicina Tropical de Sao Paulo 34 (5): 45966. https://doi.org/10.1590/s003646651992000500014.
30. Gohar AA, Galal TM, Sahar RG, Walaa SA, Atef MElS (2014) Molluscicidal Activity of the Methanol Extract of Callistemon viminalis (Sol. ex Gaertner) G.Don ex Loudon Fruits, Bark and Leaves against Biomphalaria alexandrina Snails. Iranian Journal of Pharmaceutical Research 13 (2): 505514. <Go to ISI>://WOS:000336731100016.
31. Zhong TY (2018) Study on selfassembly of snail abdominal foot gland mucus and silk fibroin / snail mucin composite adhesive. Dissertation, Suzhou University.
32. Anosh HS (2021) Preparation, Antioxidant and Antibacterial activity of Protease hydrolysate from African Snail (AchatinaFulica).Disseration, Zhejiang normal University.

33. Zhang TT, Chen CS (2013) Study on the characteristics of Baiyu snail secretion and its ability of scavenging free radicals in vitro. Chinese Journal of Food 13 (09): 2227. https://doi.org/10.16429/j.10097848.2013.09.003.
34. Liu JL, Yu JF, Wu JC, Yin JL, Wu DH (2006) Active oxygen metabolism of insects. Insect knowledge (06): 752756.
35. Xu HX, He XC, Zheng XS, Yang YJ, Lu ZX (2014) Influence of rice black streaked dwarf virus on the ecological fitness of nonvector planthopper Nilaparvata lugens (Hemiptera: Delphacidae). Insect Science 21 (4): 507514. https://doi.org/10.1111/17447917.12045.
36. Liu D, Yan SC, Cao CW, Liao YZ (2012) Effects of spinosad on detoxification and protective enzymes of Dendrolimus punctatus. Forestry science 48 (04): 6774.
37. You YC, Xie M,You MS (2013) Diversity of insect glutathione Stransferase and its mediated drug resistance. Journal of applied insects 50 (03): 831840
38. Zhang ZW, Zhao QQ, Hao X, Pan JL, Ma L (2019) Effects of avermectin and metamectin on virulence and detoxification enzyme activity of diamondback moth larvae. Journal of Northeast Forestry University 47 (05): 118122. https://doi.org/10.13759/j.cnki.dlxb.2019.05.023. https://kns.cnki.net/kcms/detail/23.1268.S.20190426.1028.046.html.
39. Bai GF, Li B, Huo SZ (2014) Effects of carbendazim on the activities of three detoxification enzymes in earthworms. Guizhou agricultural science 42 (12): 177179.
40. Zhang WL, Mou XD, Wei H, Yang YX, Xu M, Luo D, Hu YC (2016) Effects of sublethal dose of tetraacetaldehyde on the activities of AchE, GSTs and MFO in Oncomelania hupensis. plant protection 42 (06): 5862. https://kns.cnki.net/kcms/detail/11.1982.S.20161130.0945.012.html.
41. Da Silva FR, Erdtmann B, Dalpiaz T, Nunes E, Ferraz A, Martins TLC, Dias JF, Da Rosa DP, Porawskie M, Bona S, Da Silva J (2013) Genotoxicity of Nicotiana tabacum leaves on Helix aspersa. Genetics and Molecular Biology 36 (2): 269275. https://doi.org/10.1590/s141547572013005000020.
42. Yuan ZN, Cao YW, Si L, Wang DH, Guo CH (2011) The effects of nitrobenzene on the genetic toxicity in tobacco seedling leaf cells by comet assay. Molecular & Cellular Toxicology 7 (3): 291298. https://doi.org/10.1007/s1327301100358.
43. Roberts AG, Jeffrey CS, Grazyna DS, Emily ES, Santosh K, Manish BS, James RH (2023) Four Decades of Cytochrome P450 2B Research: From Protein Adducts to Protein Structures and Beyond. Drug metabolism and disposition: the biological fate of chemicals 51 (1): 111122. https://doi.org/10.1124/dmd.122.001109.
44. Xie YX, Wei JY, Chen XQ, Xiao JB (2014) Inhibition of flavonoids on acetylcholine esterase: binding and structureactivity relationship. Food & Function 5 (10): 25822589. https://doi.org/10.1039/c4fo00287c.
45. Cai SX, Xu XJ, Liu HC, Wei FH, LiuJB, Fu Y, Cao MM (2002) Comparative study on molluscicidal efficacy of niclosamide and tetraacetaldehyde. Chinese Journal of Schistosomiasis Control (04): 266269. https://doi.org/10.16250/j.32.1374.2002.04.014.
46. Yu ZH, Liu QY, Cui L, Jia XB, Zhang ZH, Jin X (2014) Preparation of ginsenoside Rb1 from ginsenoside CompoundK by Snail enzyme immobilized on Microspheres. Chinese herbal medicine 45 (21): 30923097.
47. Zhu QF,Xia CH,Fan XT,Liu RX,Tian JH,Wang L,Tang LP (1993) Studies on the fish toxic activity of tea saponin and its application Ⅴ. Hemolysis and fish toxicity of tea saponins. Tea science 01(1993):6978.
48. Shi CB, Sun XM, Zhao JY, Yuan HP (2022) Research Progress on the interaction Mechanism between tannic Acid and protein and its effect on the Physicochemical and functional Properties of protein. Science and technology of food industry 43 (14): 453460. https://doi.org/10.13386/j.issn10020306.2021070225.
49. Wang YJ, Xie KY, Zhao X, Dong KH (2011) Research Progress of Plant Tannin and its effect on Animals. Grassland and lawn 31 (04): 8286+92. https://doi.org/10.13817/j.cnki.cyycp.2011.04.018.
50. Mello S, Clelia C, Mauricio C, Jairo P, Maria LAR (2006) Physiological changes in Biomphalaria glabrata Say, 1818 (Pulmonata: Planorbidae) caused by sublethal concentrations of the latex of Euphorbia splendens var. hislopii N.E.B (Euphorbiaceae). Memorias do Instituto Oswaldo Cruz 101 (1): 38. https://doi.org/10.1590/s007402762006000100002.

51. Wang WS, Shui JH, Feng QL, Yang S, Xiang YW, Jun MY, Shi ZL, Yu HL, Luo BR, Zhang X, Hu HH, Deng ZH, Duan LP (2022) Control of the Invasive Agricultural Pest Pomacea canaliculata with a Novel Molluscicide: Efficacy and Safety to Nontarget Species. Journal of Agricultural and Food Chemistry 70 (4): 10791089. https://doi.org/10.1021/acs.jafc.1c07847.
52. Posey ND, Ma YC, Michael L, Julia D, Jeffrey AF, Vivek MP (2021) Tuning Net Charge in Aliphatic Polycarbonates Alters Solubility and Protein Complexation Behavior. Acs Omega 6 (35): 2258922602. https://doi.org/10.1021/acsomega.1c02523.

Click here to access/download;Figure;S1_raw_images.pdf

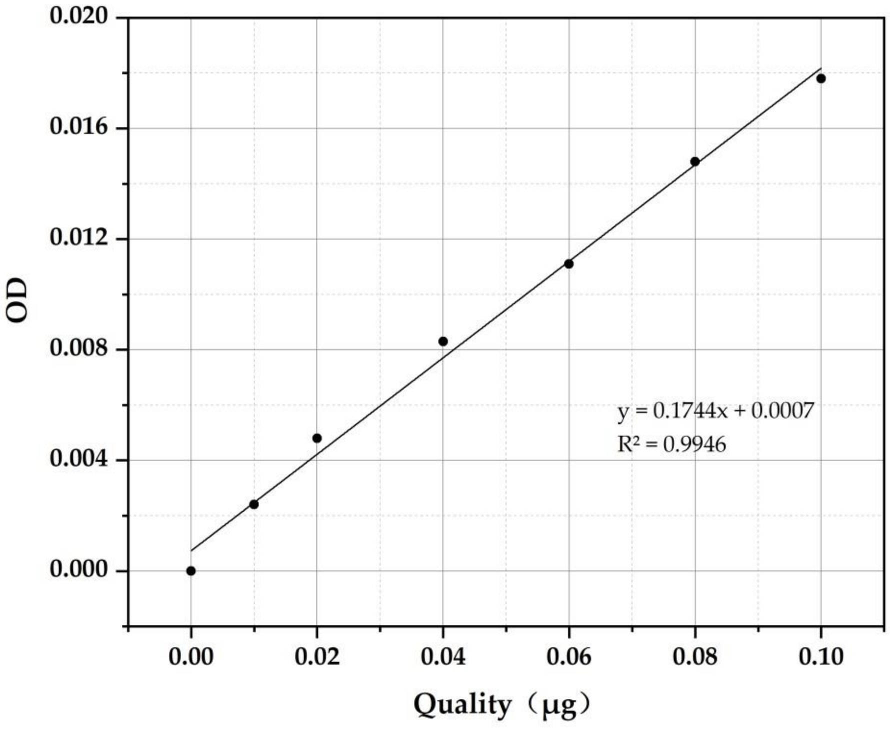

Figure 1: Rutin standard curve

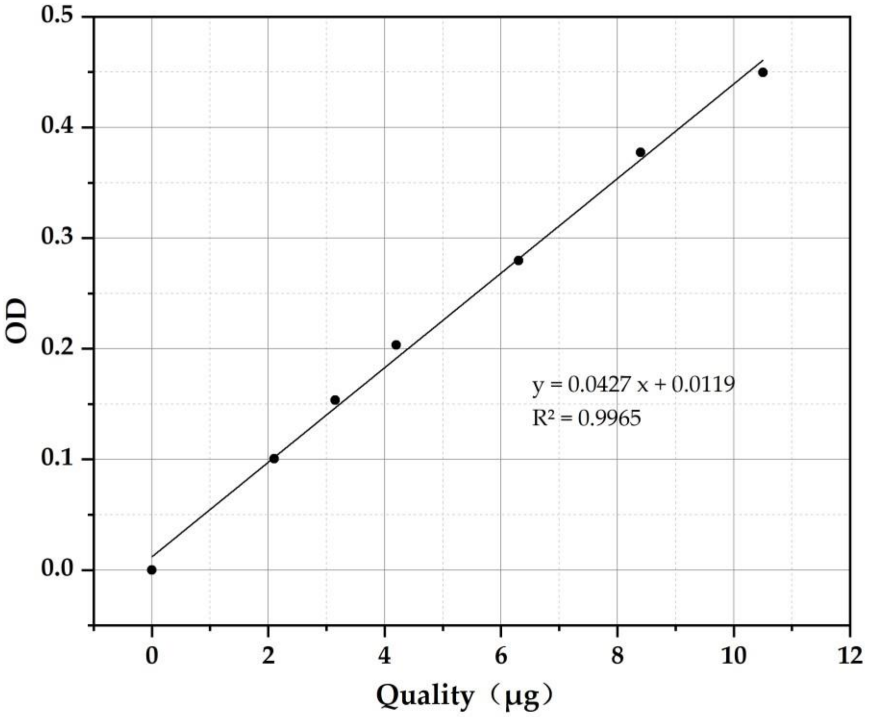

Figure 2: Ginsenoside Re standard curve

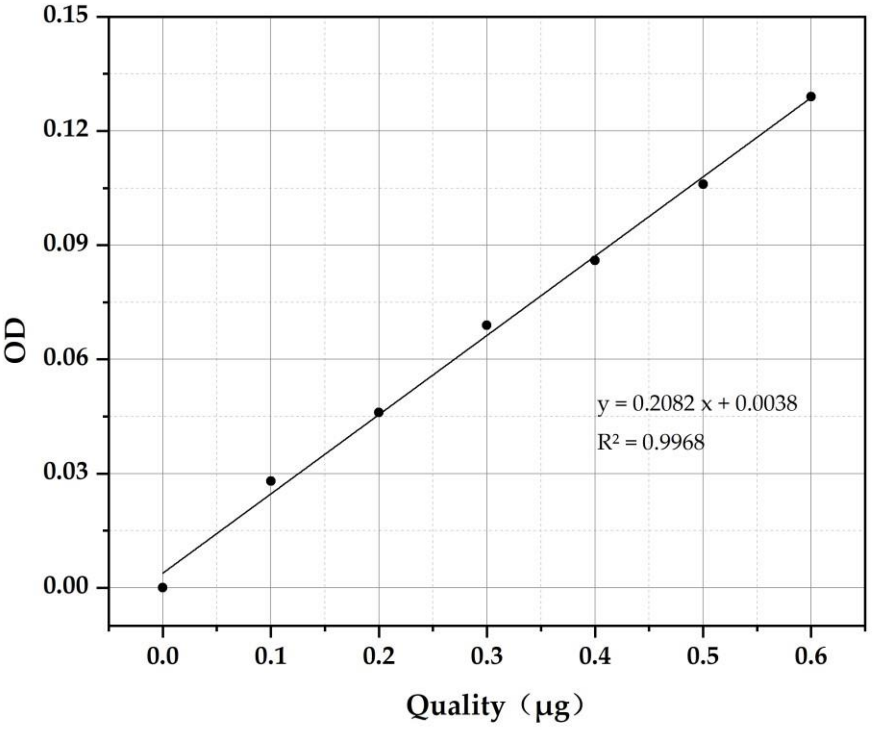

Figure 3: Gallic acid standard curve

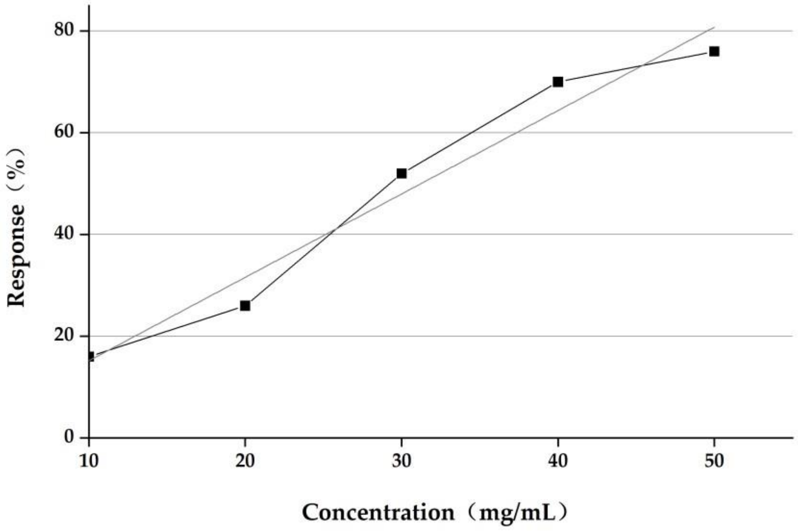

Figure 4: Concentration–response ratio of *Limax maximus* to *Ageratina adenophora* extract. Assessment response = fatality rate, expressed as a percentage of deceased *L. maximus*.

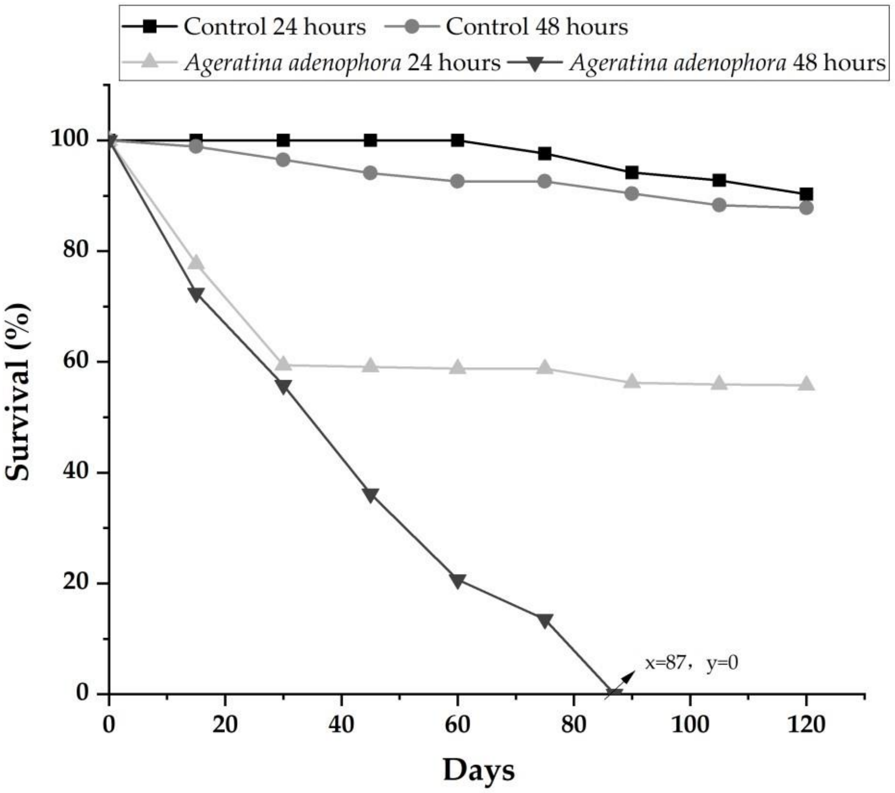

Figure 5: The survival rate of newly hatched offspring from eggs exposed to *Ageratina adenophora* extract for 24 and 48 h over a 120-day period

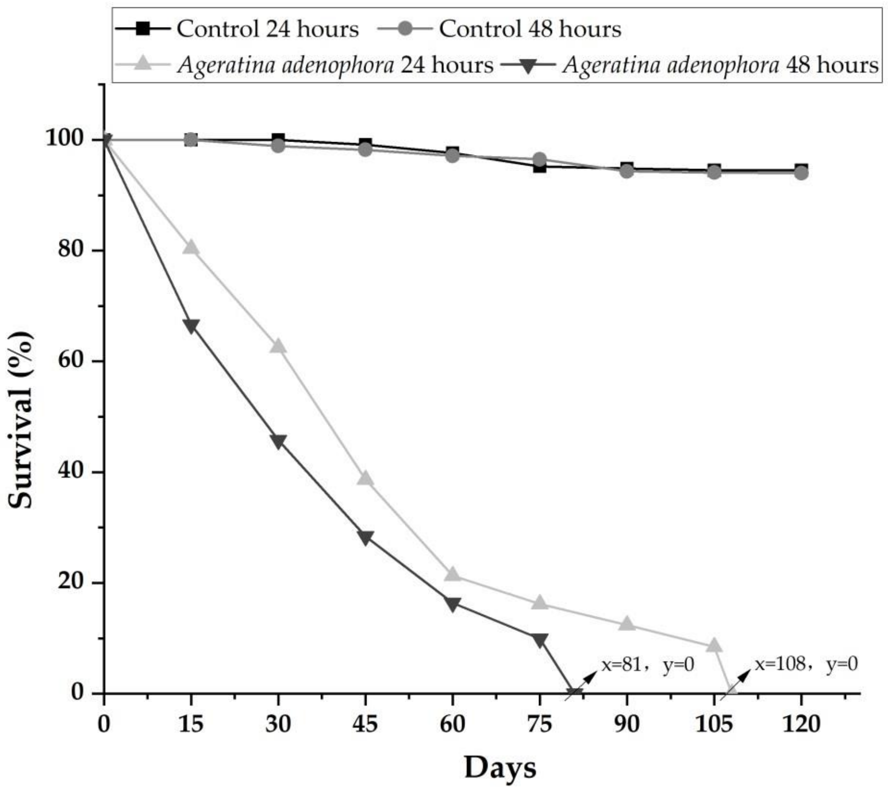

Figure 6: Observation of newly hatched slug larvae exposed to *Ageratina adenophora* extract for 24 and 48 h over a period of 120 days.

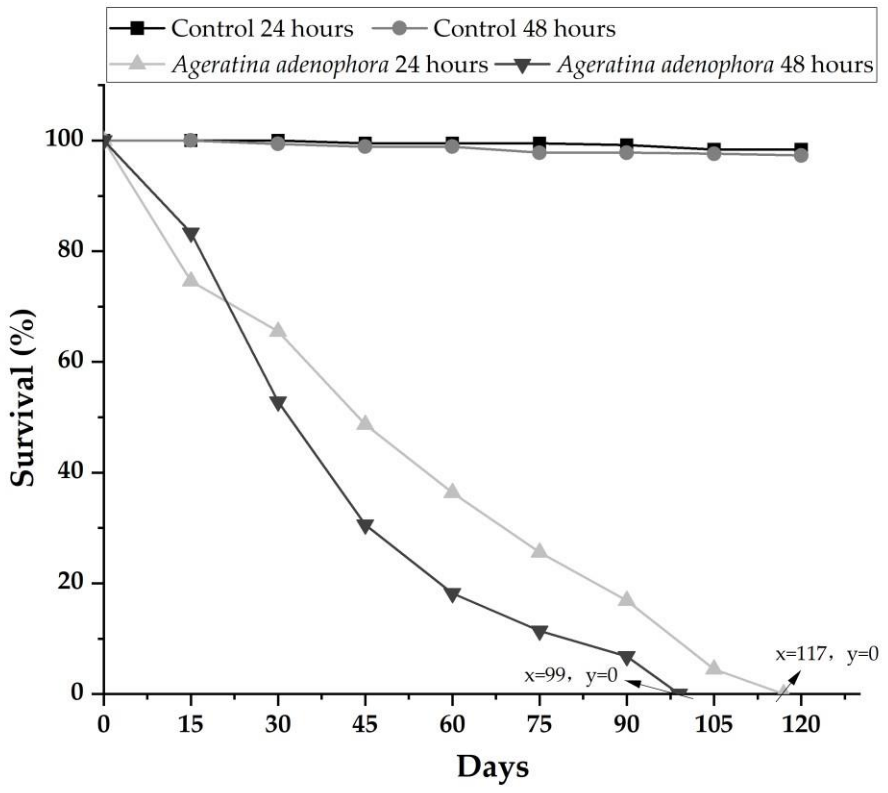

Figure 7: Survival rate of 40-day-old *Limax maximus* exposed to *Ageratina adenophora* extract for 24 and 48 h, observed over a 120 day period.

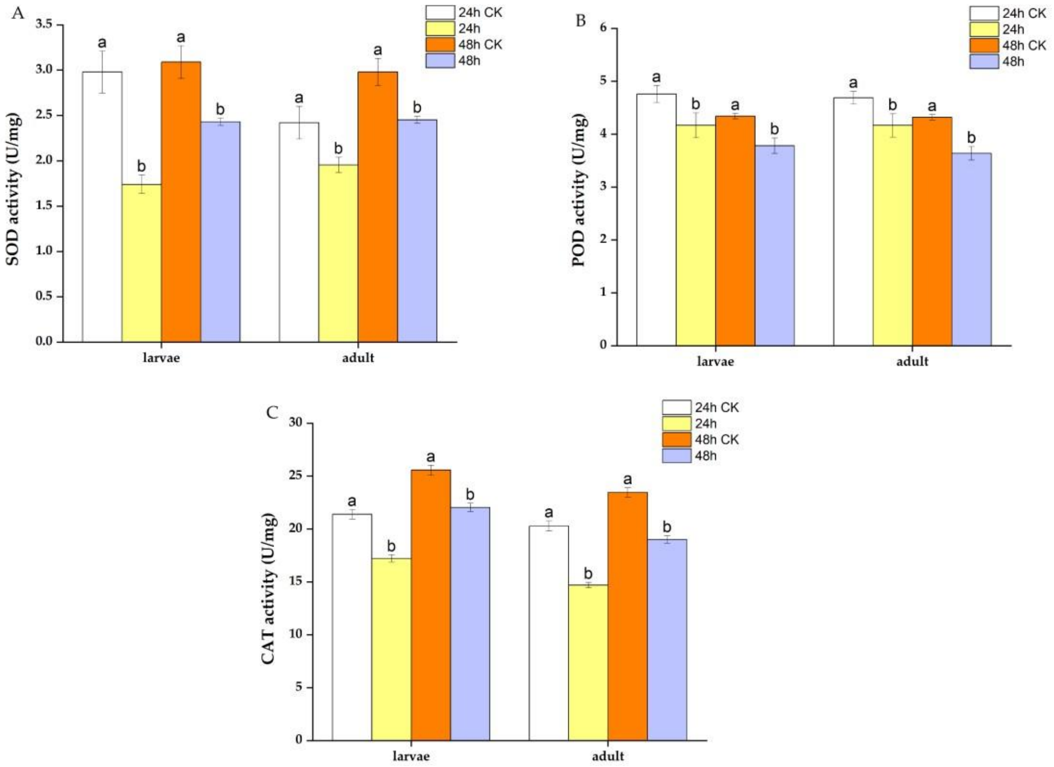

Figure 8: Protective enzyme activities of slug larvae and adults treated with LC_{50} for 24 and 48 h, respectively. The letters above the columns denote the results of multiple comparative experiments performed using Tukey tests on the enzyme activities of slug larvae and adults after LC_{50} treatment. Different lowercase letters indicate statistically significant differences.

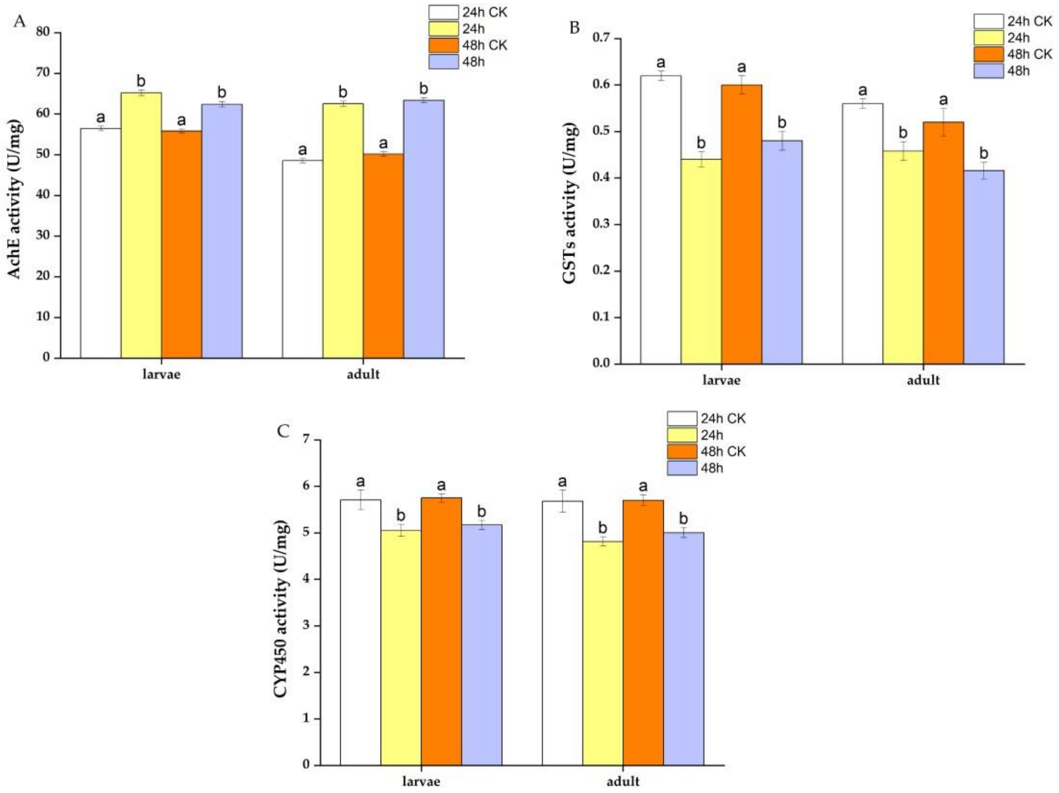

Figure 9: Detoxifying enzyme activities of slug larvae and adults treated with LC_{50} for 24 and 48 h, respectively. The letters above the columns denote the results of multiple comparative experiments

performed using Tukey tests on enzyme activities of slug larvae and adults after LC_{50} treatment. Different lowercase letters indicate statistically significant differences.
