# Supplementary material for: Insecticidal activity of Ageratina adenophora (Asteraceae) extract against Limax maximus (Mollusca, Limacidae) at different developmental stages and its chemical constituent analysis
Source: PLoS One. 2024 Apr 16;19(4):e0298668. doi: 10.1371/journal.pone.0298668 (PMC11020717; doi:10.1371/journal.pone.0298668)
Supplement: Attachment — Submitted filename: Plosone Review.docx [file pone.0298668.s002.docx]

**Abstract**

1. Please include the LC50 value specifically for 24 hours and 48 hours.
2. In my opinion, there should be two values of the LC dose, might be lower for longer exposure time.

**Introduction**

1. Expand and explain line 45-48 more the use of this plant to control mollusk species.
2. What other studies have used this plant for controlling the mollusk species. What is the specific chemical component novel from this plant which has been used for biocidal activity?

**Materials and Methods**

1. Line 56-69, please include pictures for slug and plants with its morphological identification about their species. Verification of species information required for both slug and plant.

**Results**

1. Please include a summary table for flavonoids, saponins and tannins, to enable readers for quick look at the content compositions.
2. Please include some interesting pictures showing the growth analysis, size differences between control and treatment. Line 280-286 etc

**Discussions**

1. Line 382-387, add more discussions on previous studies using plant extracts in relation to molluscicidal activity
2. Discuss the advantage of your extracts compare to chemical usage. Please include some literatures about the degradation profile on plant extracts compared to chemical treatments. Can your natural plant extracts be degraded fast in actual environment compared to chemical usage??
3. Explain more line 436-445 on chemicals harm to human and environment. What is your plant extracts applicability in industrial approach.
4. What can be the degradation profile, shelf life for your extracts, how long can your extract will be effective. Example, can your extract still be effective after keeping in solution preps for more than 1-3 months.
