# Supplementary material for: Insecticidal activity of Ageratina adenophora (Asteraceae) extract against Limax maximus (Mollusca, Limacidae) at different developmental stages and its chemical constituent analysis
Source: PLoS One. 2024 Apr 16;19(4):e0298668. doi: 10.1371/journal.pone.0298668 (PMC11020717; doi:10.1371/journal.pone.0298668)
Supplement: Attachment — Submitted filename: Response to Reviewers.docx [file pone.0298668.s004.docx]

**Dear Editor and Reviewers,**

**Thanks very much for taking your time to review this manuscript. I really appreciate all your comments and suggestions! Please find my itemized responses in below and my revisions/corrections in the re-submitted files**

**SUGGESTIONS FROM EDITOR**

**The introduction part is poorly written, and several details are missing in the current form. Please add information about current control methods and their disadvantages, then write about alternative biological control methods. Only then will the reader understand clearly; otherwise, they will be confused.
Line 60: typo error
Line 66: Why did you dry plant materials at 60°C for 24 hours? because at 60 oC, most of the volatile compounds will degrade, so please check it.
Line 66: Please add the made and made of the mechanical grinder.
Why have you selected only distilled water for extraction? What about other solvents?
Figures 1–9: Please change them and add high-quality images.**

**Academic Editor:**

1. I have perfected the introduction of the manuscript, adding the current control methods and shortcomings, and explaining the alternative biological control methods, and why plant extracts are selected for control.
2. Line 60: typo error (Modified)
3. Line 66: Why did you dry plant materials at 60°C for 24 hours?

I chose to dry at 60℃ for 24 hours because I referred to someone else's method. Most plant extracts need to be dried before they are prepared. Plant extracts mainly play a role in secondary metabolites, which can only be extracted by heating or even evaporation, and the extracts in this study still have obvious toxic effect on *Limax maximus*.

4. Line 66: Please add the made and made of the mechanical grinder. (Added)
5. Why have you selected only distilled water for extraction? What about other solvents?

First of all, distilled water is selected because its influencing factors are small and suitable as a control, and pre-experiments have been done to confirm that it has no toxic effect on *Limax maximus*, and other solvents may have a better effect. This is a point that needs to be improved in our research in the future. Thank you very much for your suggestion.

6. Figures 1–9: Please change them and add high-quality images. (Changed)

**Reviewer #1:**

**Abstract**

1. **Please include the LC_50_ value specifically for 24 hours and 48 hours**

The LC_50_ value in our study was calculated by virulence test. 24 h and 48 h were the treatment time set by the extract with LC_50_ concentration, so there was only one LC_50_ value.

2. **In my opinion, there should be two values of the LC dose, might be lower for longer exposure time**

LC_50_ is a semi-lethal concentration, the specific meaning is that in the acute toxicity test of animals, the concentration of poison that makes half of the animals killed is generally only one value, and the virulence test is carried out at the same time, the difference is that the concentration of dilution is different.

**Introduction**

1. **Expand and explain line 45-48 more the use of this plant to control mollusk species**

At present, the example of using *Ageratina adenophora* to control molluscs is still very rare. the purpose of using *Ageratina adenophora* extract on *Limax maximus* in our study is to explore whether this extract has killing effect on *Limax maximus*. in order to develop a new botanical pesticide.

2. **What other studies have used this plant for controlling the mollusk species. What is the specific chemical component novel from this plant which has been used for biocidal activity?**

No other studies have used this plant to control molluscs, and the special chemical components we have identified are flavonoids, saponins and tannins, all of which have bactericidal activity. however, we do not rule out the fact that other undetected chemical components also have killing activity, which is also a direction of our future research.

**Materials and Methods**

1. **Line 56-69, please include pictures for slug and plants with its morphological identification about their species. Verification of species information required for both slug and plant**

We have added pictures of *Limax maximus* and *Ageratina adenophora* to the manuscript, because we have given detailed species names, and this study does not belong to the taxonomic category, and the corresponding species can be found well according to the species names.

**Results**

1. **Please include a summary table for flavonoids, saponins and tannins, to enable readers for quick look at the content compositions.**

We have attached a summary table to make the data more intuitive.

2. **Please include some interesting pictures showing the growth analysis, size differences between control and treatment. Line 280-286 etc**

We added a growth comparison chart to the **Supporting Information Captions**.

**Discussions**

1. **Line 382-387, add more discussions on previous studies using plant extracts in relation to molluscicidal activity**

We added some discussions about the use of plant extracts to control molluscs.

2. **Discuss the advantage of your extracts compare to chemical usage. Please include some literatures about the degradation profile on plant extracts compared to chemical treatments. Can your natural plant extracts be degraded fast in actual environment compared to chemical usage??**

Chemical agents are often accompanied by 3R problems, and there are few articles about the degradation of plant extracts compared with chemicals. At present, the use of plant extracts as a new chemical is still in the experimental stage. Field experiments and degradation analysis have not yet been carried out. In order to really apply it to practical control, we will speed up our research to ensure compliance with the principles of the use of pesticides. Thank you for your suggestions.

3. **Explain more line 436-445 on chemicals harm to human and environment. What is your plant extracts applicability in industrial approach.**

We have explained the harm of chemicals to human beings and the environment, as well as the convenience of our extracts in factory production.

4. **What can be the degradation profile, shelf life for your extracts, how long can your extract will be effective. Example, can your extract still be effective after keeping in solution preps for more than 1-3 months.**

The degradation of our extract has not been studied yet, which is an important indicator for us to apply it to field experiments. Our extract can still be effective after 1-3 months of preservation, which has been explained in the manuscript.

**Reviewer #2:**

We modified some sentence patterns and phrases according to the requirements of the reviewer#2.

**We would like also to thank you for allowing us to resubmit a revised copy of the manuscript.**

**We hope that the revised manuscript is accepted for publication in the Plos One.**
