# Supplementary material for: Insecticidal activity of Ageratina adenophora (Asteraceae) extract against Limax maximus (Mollusca, Limacidae) at different developmental stages and its chemical constituent analysis
Source: PLoS One. 2024 Apr 16;19(4):e0298668. doi: 10.1371/journal.pone.0298668 (PMC11020717; doi:10.1371/journal.pone.0298668)
Supplement: Attachment — Submitted filename: Comments@2024.docx [file pone.0298668.s005.docx]

| **Sections** | **Comments** |
| --- | --- |
| Introduction | 1. There is additional reasoning given for the studies, especially on the parasite factors. 2. Changes has been done and accepted |
| Materials and Methods | 1. Morphological details were given and changes acceptable. 2. Pictures are acceptable |
| Results | 1. Standard curve and details are acceptable and suitable for data representations 2. Compositions of chemicals acceptable |
| Discussions | 1. Discussions are acceptable and additional points is more relevant |
