# Supplementary material for: Insecticidal activity of Ageratina adenophora (Asteraceae) extract against Limax maximus (Mollusca, Limacidae) at different developmental stages and its chemical constituent analysis
Source: PLoS One. 2024 Apr 16;19(4):e0298668. doi: 10.1371/journal.pone.0298668 (PMC11020717; doi:10.1371/journal.pone.0298668)
Supplement: Attachment — Submitted filename: Response to Reviewers.docx [file pone.0298668.s006.docx]

**Dear Editor and Reviewers,**

**Thanks very much for taking your time to review this manuscript. I really appreciate all your comments and suggestions! Please find my itemized responses in below and my revisions/corrections in the re-submitted files**

**SUGGESTIONS FROM EDITOR**

**We have uploaded our figure files to the Preflight Analysis and Conversion Engine (PACE) digital diagnostic tool to ensure that our pictures meet the requirements.** **We added the scale bar to Fig 1 and the clarity of Fig 3, 4, 5, 6 and 7 to make it look clearer.**

**Finally, we checked our reference list to make sure that our reference list were complete and accurate and not rejected.**

**We would like also to thank you for allowing us to resubmit a revised copy of the manuscript.**

**We hope that the revised manuscript is accepted for publication in the Plos One.**
